# A Biologic and Physical Characterization of an Injectable Amniotic Membrane Designed for Treating Diabetic Foot Ulcers

**DOI:** 10.3390/bioengineering11111087

**Published:** 2024-10-30

**Authors:** Kimberly Velarde, Audrey Arvonen, Tatyana Gonzalez, Robert B. Diller

**Affiliations:** 1 Amnio Technology, LLC., 22510 N. 18th Dr., Phoenix, AZ 85027, USA; kvelarde@amniotechnology.com (K.V.); aarvonen@amniotechnology.com (A.A.); tgonzalez@amniotechnology.com (T.G.); 2 Department of Biological Sciences, Northern Arizona University, Flagstaff, AZ 86011, USA

**Keywords:** amniotic membrane, novel birth tissue injectable, wound healing, diabetic foot ulcer

## Abstract

Introduction: Globally, the health and quality of life of millions of people are negatively affected by diabetic foot ulcers (DFUs). To treat these chronic wounds, a novel injectable drug for closing DFUs composed of micronized amniotic membrane was developed. This new therapeutic drug for wound repair expands on traditional allograft therapies by allowing extracellular matrix proteins, growth factors, and cytokines to reach wound anatomies in DFUs that are difficult to treat. The aim of this study was to evaluate the components of the injectable drug. Methods: Liquid chromatography with tandem mass spectrometry and a Quantibody^®^ human cytokine array were conducted to identify and characterize growth factors and proteins known to contribute to wound healing. In addition, hyaluronic acid was quantified and compared between the injectable and human amniotic fluid using a hyaluronan enzyme-linked immunosorbent assay. Cell proliferation, migration, angiogenesis, and viability were evaluated to assess the performance of the novel injectable in vitro. The rheometric properties of the product were evaluated by assessing it pre- and post-injection through a 22-gauge needle to measure the viscosity using a shear- and temperature-dependent viscosity protocol. Results: Liquid chromatography with tandem mass spectrometry and Quantibody^®^ human cytokine array revealed growth factors and proteins imperative for wound healing. The quantified hyaluronic acid was compared between the injectable and human amniotic fluid, resulting in a statistically significant difference, with higher protein concentrations found in the injectable. In vitro qualitative and quantitative analysis confirmed an increase in cell viability, proliferation, and migration when treated with the drug. An evaluation of the rheometric properties of the injectable drug after passing through a 22-gauge cannula presented no alterations to the biologic drug. Conclusions: Collectively, these data present the potential of a novel injectable drug for the treatment of DFUs.

## 1. Introduction

### 1.1. Diabetes Mellitus: Diabetic Foot Ulcers

Diabetes mellitus (DM) is a debilitating chronic disease that affects the quality of life of millions of people. It is predicted that, by 2030, approximately 552 million people worldwide will be diagnosed with DM and, by 2045, up to 783 million people will succumb to this disease [1,2]. Patients with DM are associated with numerous cardiovascular and neurological comorbidities, which can lead to serious outcomes like peripheral vascular diseases, renal failure, retinopathy, neuropathy, blindness, limb amputations, and death [2,3]. For instance, the lower limbs of diabetic patients can develop peripheral vascular disease and neurological conditions that lead to amputation of lower extremities, primarily caused by diabetic foot ulcerations [4]. Approximately 25% of diabetic patients will develop DFUs, with only 46% of DFUs healing, resulting in a 25% incidence rate of limb amputation [2,5]. It is estimated that the mortality rates associated with DFUs are 5% in the first 12 months, continuing to rise to 42% within 5 years [6].

Many factors contribute to a slow DFU healing process. The most common diabetic complications affecting the healing process are a combination of hyperglycemia, peripheral vascular diseases, and neuropathy [7]. Hyperglycemia also plays a role in diabetic neuropathy by damaging the small blood vessels that supply blood flow to peripheral nerves [8]. Diabetes causes motor, sensory, and autonomic fiber denervation, affecting the cutaneous autonomic nerve fibers responsible for regulating the dermis and blood vessels [9]. Denervated wounds like DFUs interfere with the healing process, causing a delay in re-epithelialization [10]. As a result, blockage of blood vessels in the lower extremities can interfere with the blood flow by decreasing oxygen levels, leading to mild hypoxia and increasing the chances of developing peripheral arterial disease in diabetics [7].

### 1.2. Process of Wound Healing

**Figure 1:**
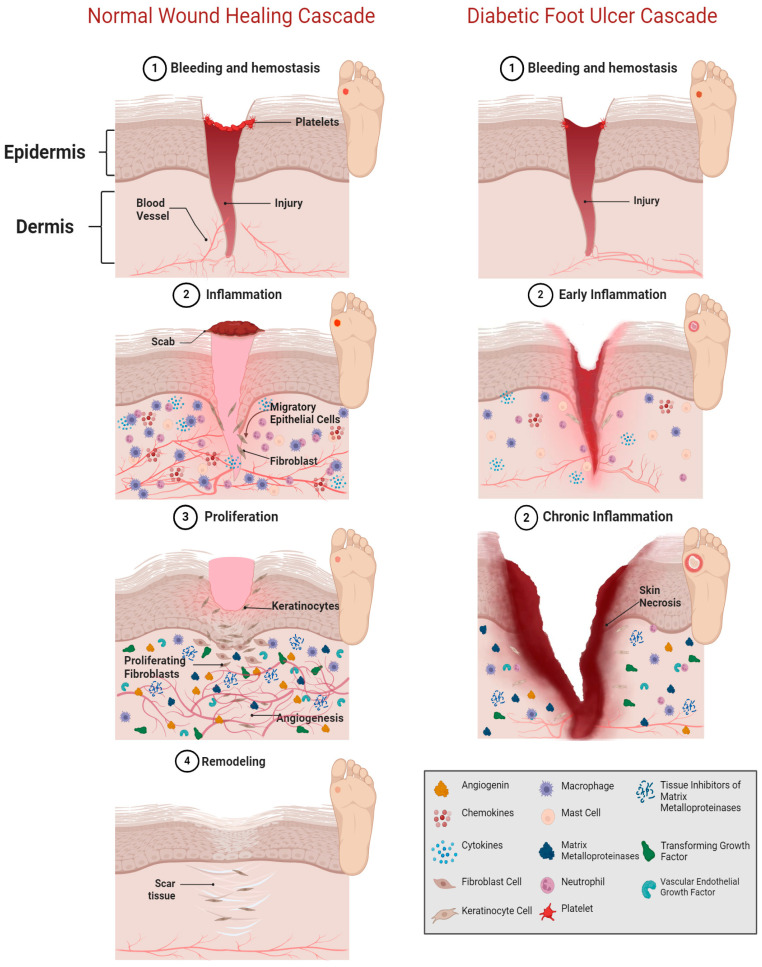
Comparison of the wound healing cascade in a normal wound versus a diabetic foot ulcer. Shown on the left, a normal wound healing cascade completes all four stages of healing: hemostasis, inflammation, proliferation, and remodeling. Higher quantities of the extracellular matrix proteins, chemokines, growth factors, immune cells, and cytokines required for wound healing can be seen in a normal wound. As the wound healing continues, an overlap of the inflammatory and proliferation stage promotes angiogenesis and the proliferation of endothelial cells. The large vascular network enhanced by angiogenesis aids in wound closure and scar tissue formation. On the contrary, as shown on the right, a diabetic foot ulcer lacks the proper vasculature to deliver the necessary proteins needed to close a wound and remains in a chronic inflammatory state, despite the available interventions. As a result, the ulcer continues to worsen, leading to tissue necrosis. (Image created with BioRender.com).

In healthy individuals, physiological wound healing can be broken down into four overlapping stages that prevent infection and remodel the damaged tissue: hemostasis, inflammation, proliferation, and remodeling [11,12]. These four stages are crucial, as the longer a wound remains inflamed, the more likely it is to become chronic, painful, or have increased scarring [13]. As seen in Figure 1, the activation of multiple pro-inflammatory cytokines and growth factors is required to initiate and complete the wound healing process. In the first stage of tissue restoration, a state of hemostasis must be achieved to control the bleeding of damaged blood vessels and prevent infection. This is achieved by a clotting cascade of platelets exposed to collagen proteins, causing the aggregation of a fibrin matrix to form a clot [14]. Clot formation provides stability to the ECM, as platelets recruit and promote other immune cells to perform their function via signaling molecules [15]. Once the bleeding has been controlled, fibronectin starts promoting cell adhesion, migration, proliferation, and apoptosis, to begin the second stage [16]. The role of fibronectin is crucial in all four stages, as it aids in the arrangement and formation of the ECM for wound and connective tissue repair [17].

Next is the inflammation stage, where chemokines help initiate cell recruitment of neutrophils, macrophages, and mast cells. Together, these cells help to clear cell debris and foreign bodies, and degrade the ECM through phagocytosis, thus releasing inflammatory cytokines to promote tissue regeneration [18]. Furthermore, chemokines help activate pro-angiogenic molecules to aid in endothelial cellular migration, proliferation, and differentiation [19]. Factors that contribute to DFUs remaining in the inflammatory phase include the inability to clear cell debris, promote cells to proliferate, and establish a healthy vascular network. The longer the wound remains inflamed, the higher the likelihood of it becoming necrotic.

In the third stage of wound healing, fibroblasts, macrophages, and endothelial cells work together to promote angiogenesis and ECM remodeling. Angiogenesis involves angiogenic growth factors like transforming growth factor (TGF)-β, TNF-α, vascular endothelial growth factor (VEGF), PDGF, fibroblast growth factor (FGF), angiogenin, and angiopoietin-1, together with anti-angiogenic growth factors like angiostatin, tissue inhibitor of metalloproteinases 2 (TIMP-2), Interferon (IFN)-α, and IFN-β to form new capillaries from already established vasculature [20,21]. Angiogenesis is also regulated by angiogenin, to stimulate endothelial cell proliferation and migration to the wound site, while contributing to the remodeling of the ECM [22]. If angiogenesis is impaired, the delivery of cytokines and chemokines is suppressed, leading to an increase in hypoxia, oxidative stress, and further delays in progressing to the proliferation stage [23].

The degraded ECM from the second stage of wound healing is restored during the remodeling stage and can take from one week to six months to achieve complete closure [11,24]. The ECM is a multi-cellular network composed of collagen, proteoglycans, glycosaminoglycans, glycoproteins, and elastin [11,25]. Together these proteins provide structure to the tissue by forming a scaffold that promotes cellular adhesion, migration, growth, and differentiation [26]. As proliferation transitions into remodeling, the fibronectin, fibrin, hyaluronic acid (HA), and other structural components replace the degraded ECM [27]. For instance, unorganized collagen III is an immature collagen protein created from fibroblasts during the proliferation stage and is broken down and replaced by collagen I in the remodeling stage to form a more uniform cross-linked structure [14,28]. Failure to replace collagen III with collagen I can severely increase scar tissue formation in DFUs [29].

As collagen fibers aid in the structure of new tissue, simultaneously, HA contributes to cellular proliferation, differentiation, adhesion, and migration during tissue regeneration [29]. Eventually, the amount of HA and fibronectin decreases, as the wound no longer needs to be provided with cellular adhesion, and is then replaced by mature ECM [17,30]. Finally, the remodeling stage comes to an end once fibroblasts reorganize the collagen matrix to form scar tissue and provide structural support, which can take several months to achieve [19]. Most healthy individuals can complete these stages without medical interventions, despite a wound’s severity. For diabetics, many contributing factors can easily impede the process of wound healing, making it difficult to live a normal life.

### 1.3. Quality of Life: Living with Diabetes

These very complex stages of hemostasis, inflammation, proliferation, and remodeling are very difficult to achieve in diabetics. Many health complications from comorbidities can cause certain treatments to be ineffective in healing DFUs; however, without treatment, the onset of tissue necrosis, sepsis, and amputation can occur. Ultimately, an amputation is a traumatic event that can alter the lifestyle of patients by causing them to undergo psychological, physical, and economic distress during the treatment of DFUs [31,32]. As the severity of the foot ulceration progresses, the patient’s mobility begins to become restricted, negatively affecting everyday activities. As a result of this disease, the quality of life continues to decline, despite preventative measures and current medical treatments.

### 1.4. Current Treatments for Diabetic Foot Ulcers

The generalized wound management treatment of diabetic foot ulcers is to increase the healing process, ultimately removing the infected tissue from the wound bed surgically to allow re-epithelialization to occur. Standard of care (SOC) is commonly provided for DFUs, as it consists of wound cleansing using sterile normal saline solution, moist dressing, hydrogel, and wound offloading using an offloading device [33]. Recent treatments such as skin grafts and bioengineered tissues have been used as a reconstructive method, where wound healing is promoted by releasing ECM components, growth factors, and cytokines [6]. The mechanism by which skin grafts and bioengineered tissues release wound healing components is not fully understood; nonetheless, an analysis of studies focused on skin graft and bioengineered skins concluded that these treatments should not be used on their own, but alongside SOC [34]. Other treatments like adjuvant therapies consist of debridement agents, wound dressings, topical agents, oxygen therapy, negative pressure, and cellular therapies [6]. Despite the current treatments available, finding the appropriate option for each individual diabetic can be time consuming and can cause both psychological and economic distress.

For wounds like DFUs, both intramuscular and subcutaneous injectable treatments are most beneficial as they allow for immediate delivery to localized regions. For example, injectable hydrogels are used as a bandage to improve wound healing. This is achieved when the injectable hydrogel fills the wound bed, allowing for direct drug delivery [35]. Despite these advantages, hydrogel injection therapies possess disadvantages that can also cause high cell death, poor engraftment, lack of control over the spacing between cells or cell density, and secondary infections, they can also be complex procedures with high treatment costs, and may require debridement [36]. For this purpose, an injectable composed of micronized amniotic membrane capable of delivering the necessary components to promote closure of DFUs was developed.

### 1.5. Creation of an Injectable Amniotic Membrane (IAM)

Amniotic membrane (AM) has been used to heal chronic wounds since the second half of the 20th century, as it is an ideal 3D scaffold for cellular activity [37]. Presently, AM is used in numerous applications for human pathologies, ranging from wound healing to disorders of the ocular surface, oral cavity, skin, stomach, larynx, genito-urinary tract, diabetic neurovascular ulcers, postsurgical wounds, and much more [38,39]. AM is seen as an exemplary candidate for wound management, as it possesses anti-inflammatory, anti-scarring, bacteriostatic, and non-immunogenic properties that assist in re-epithelialization and angiogenesis [37,40,41]. Multiple properties of AM are associated with the synthesis and release of cytokines and signaling molecules, such as TNF-α, TGF-α and β, basic fibroblast growth factor (bFGF), epidermal growth factor (EGF), keratinocytes growth factor (KGF), interleukins (IL-4, IL-6, IL-8), natural metalloprotease inhibitors, defensins, prostaglandins, and more [42,43]. Patients suffering from DFUs would greatly benefit from AM, which can be directly applied into the wound bed to immediately begin the stages of healing.

As previously mentioned, amniotic tissue allografts have complex and multifaceted modes of action. Amniotic tissue contains a heterogeneous mix of ECM proteins, cytokines, and growth factors, individually known to facilitate wound healing and tissue regeneration [44]. To take advantage of the potential of amniotic membranes, a liquid parenteral dosage format was constructed to facilitate the liquid allograft in reaching and treating wounds with irregular surfaces, sinus tracts, undermining, and other wound anatomies that present challenges for intact amniotic membrane allografts to interact with completely. Administering a micronized form of amniotic membrane directly to areas surrounding the wound allows direct contact with the impaired tissue. Unlike current available treatments, this novel injectable is actively undergoing a phase II clinical trial for treating lower extremity ulcers (chronic, non-traumatic wounds greater than four weeks in duration).

**Figure 2:**
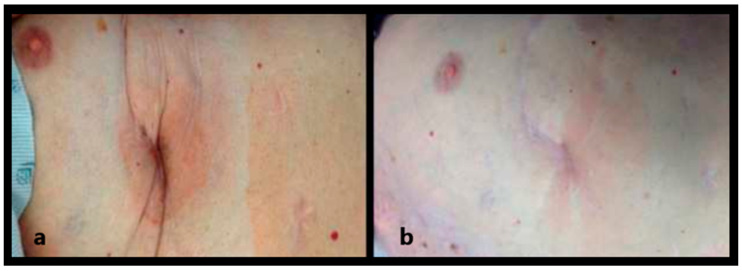
Thoracotomy scar pre (**a**) and post (**b**) IAM treatment. IAM was directly injected into the surrounding superior, inferior, medial, and lateral sections of the thoracotomy scar. Reprinted with permission from Ref. [45]. 2014, Elsevier.

A study conducted by Hemphill et al. exhibited, for the first time in a human subject, the use of an amniotic membrane matrix injectable to remodel fibrotic scar tissue caused by late-stage fibrosis after failure to heal using conventional therapeutic interventions [45]. In their study, a patient with a history of ischemic cardiomyopathy was subjected to several surgeries, resulting in a decline in quality of life. The patient was treated with 2 mL by injecting directly into the surrounding sites of the thoracotomy scar. After one treatment, the patient’s pain decreased dramatically, and after two more treatments, the patient was pain-free, and the remodeling of the thoracotomy scar had been achieved (see Figure 2). The astounding results show the effectiveness of an amniotic membrane matrix injectable in promoting wound closure in vivo.

This study’s aim was to present a brief overview of the composition of this micronized amniotic membrane injectable, currently in a phase II clinical trial, by carrying out a series of assays. Liquid chromatography with tandem mass spectrometry (LC-MS/MS) analysis and a Quantibody human cytokine array were conducted for the characterization of the multiple proteins found in IAM and their involvement in wound healing. The concentration of hyaluronic acid in IAM was of interest, as this ubiquitous glycosaminoglycan (GAG) is known to assist in tissue repair. To quantify the amount of HA found in IAM compared to human amniotic fluid, a hyaluronan enzyme-linked immunosorbent assay was performed. Furthermore, qualitative and quantitative analysis of the effects of IAM on cell proliferation, viability, and migration of human dermal fibroblast adult (hDFa) cells was evaluated. The in vitro angiogenic effects of IAM on HUVEC tube formation were assessed. In addition, the rheometric properties of the IAM were examined to determine the viscosity and rheometric behavior of the fluid through a 22-gauge cannula. A USP <507> Protein Determination, Method II Bicinchoninic Acid assay was conducted to assess the stability of the protein content of the IAM after 2 years from manufacture. Together, these assays provide initial information on the biologic, chemical, and physical components of IAM for use as a novel injectable drug for the treatment of DFUs.

## 2. Materials and Methods

Three total lots of IAM used for testing were created in a laboratory following validated manufacturing batch records. For the creation of the IAM, all placental tissues were donated following cesarean section births, and consent was obtained prior to collection for the tissues to be used for research. The donors had to complete an extensive medical and social history investigation to be considered for donation. All tissues were screened for bacteria at collection, and blood was taken to ensure there was no viral contamination to meet the Food and Drug Administration (FDA) and the American Association of Tissue Bank (AATB) criteria. No fetal tissues were used in processing the IAM, and the placental tissues were only collected if there were no complications at the time of delivery.

The IAM treatment was developed as a liquid and marketed as a Human Cell Tissue Product (HCT/P), as set forth in 21 CFR part 1271 prior to 2020. This liquid human amniotic tissue allograft contains micronized amnion membrane and elements obtained from amniotic fluid (extracellular matrix and protein) suspended in an injectable electrolyte solution containing dimethyl sulfoxide (DMSO). During manufacturing, the amniotic membrane was micronized and mixed with an injectable electrolyte solution for a final concentration of 1 cm^2^/mL. The composition of any additives in the electrolyte solution and the details of the manufacturing processing of the IAM are proprietary.

### 2.1. Characterization of the Amniotic Fluid Component of IAM

Mass spectrometry analysis was conducted by the Arizona State University (ASU) Core Mass Spectrometry lab to evaluate the amniotic-fluid-derived portion of the drug substance in IAM. First, the amniotic fluid was prepared for mass spectrometry analysis by centrifugating for 10 min at 1450 rpm and 4 °C. The supernatant was decanted, and the pelleted solids were resuspended in Plasmas Lyte A and aliquoted into cryovials. Samples were stored at −80 °C until they were ready to ship for LC-MS/MS analysis. The following description of the methods used for LC-MS/MS analysis was provided by the ASU Core Mass Spectrometry lab.

Samples were centrifugated at 4 °C on the highest setting for 20 min. The supernatant was discarded, and samples were centrifugated again at 4 °C on the highest setting for 20 min. The supernatant was discarded, and the pellet was dissolved in 12.5 µL of 8 M urea solution. This amount was found appropriate, as higher volumes could reduce the enzyme activity of trypsin. A total of 87.5 µL of 100 mM of triethylammonium bicarbonate was added to the sample. Next, 5 µL of 200 mM of Tris (2-carboxyethyl) phosphine was added, and the sample was incubated at 55 °C for 1 h. After incubation, the sample was removed from the heat and allowed to cool to room temperature. After the sample had cooled, 5 µL of 375 mM of iodoacetamide was added to the sample and incubated for 30 min in the dark at room temperature. Lastly, 2.5 µL of trypsin (2.5 µg per 100 µg of protein) was added to the sample and incubated at 37 °C overnight.

The following day, the PiERce C18 Spin Column procedure was performed for further sample purification (Thermo Fisher). The preparation of the activation solution consisted of 50% methanol/50% water, and 400 µL was added per sample. The sample buffer was made with 2% trifluoroacetic acid in 20% acetonitrile/80% water. For the buffer, 1 µL was added per 3 µL of sample. The equilibration solution was prepared using 0.5% trifluoroacetic acid in 5% acetonitrile/95% water. For each sample, 400–800 µL of equilibration solution was added, dependent upon the amount and type of contaminants present in the sample. Lastly, the elution buffer was made with 70% acetonitrile/30% water, and 40 µL was added to each sample. Mass spectrometry analysis of the samples was conducted using a Thermo Orbitrap Fusion Lumos Tribrid Mass Spectrometer (Lumos) with an UltiMate 3000 RSLCnano System. The proteins in the amniotic fluid solids were identified by submitting the raw tandem mass spectral files to the SEQUEST HT and matching the peptides to known protein sequence databases.

### 2.2. Quantibody Protein Characterization of the IAM

A Quantibody Human Cytokine Array (QAH-CAA-4000, RayBiotech, RayBiotech, Norcross, GA, USA) was conducted by RayBiotech on the IAM, to accurately detect the concentration of 200 human inflammatory factors, growth factors, chemokines, receptors, and cytokines. Three undiluted IAM samples were tested in duplicate, along with the product vehicle agents. IAM samples were eluted at room temperature for 24 h and then spun at 1400× *g* for 10 min. The supernatant was aliquoted into cryovials and frozen at −80 °C. The vehicle agent was prepared and then frozen at −80 °C. All samples were sent to RayBiotech for analysis. Imaging was performed using a laser scanner equipped with a Cy3 wavelength, the data were extracted, and a Q-Analyzer analysis was performed.

### 2.3. Hyaluronic Acid Content in the IAM

Important proteins like hyaluronic acid have been found in human amniotic fluid and membrane. For this purpose, the concentration of HA in the IAM was quantified in parallel with the amniotic fluid for comparison. The HA was measured by following the manufacturer’s instructions of a Hyaluronan enzyme-linked immunosorbent assay (HA-ELISA) (K-1200, Echelon Biosciences, Salt Lake City, UT, USA). For this test, three IAM samples were prepared by allowing the amniotic membrane and fluid components to elute in the background solution at room temperature for 20 h, before filtering through a 0.22 µm syringe filter. Next, three human amniotic fluid samples were filtered through a 0.22 µm syringe filter. The absorbance was determined at 405 nm using a microplate reader (Synergy HTX, Biotek Instruments Inc., Winooski, VT, USA) and the average concentrations of HA in IAM and amniotic fluid were interpolated based on the HA standard curve. An unpaired t-test was conducted using GraphPad Prism software, version 9.4.1 to analyze the difference between the IAM and human amniotic fluid.

### 2.4. Human Dermal Fibroblast Cell Proliferation

The effects of the IAM on the cellular proliferation of hDFa cells (ATCC) were assessed using a CyQUANT^®^ Cell Proliferation Assay kit (C7026, Life Technologies, Carlsbad, CA, USA). Cell-culture-treated 96-well plates were used to seed hDFa cells at a cell density of 5000 cells/well in Dulbecco’s modified Eagle’s medium (DMEM) supplemented with 10% fetal bovine serum (FBS) and 0.01% Pen/Strep (15-140-122, Gibco). hDFa cells were incubated at 37 °C with 5% CO_2_ for 20 ± 2 h to allow cell adherence prior to treatment. Next, the cell media were aspirated and treated with 200 µL/well of either the IAM, supplemented DMEM (complete media as the positive control), or serum-free DMEM (basal media as the negative control), and incubated for 48 ± 2 h in an incubator set at 37 °C with 5% CO_2_. After incubation, cells were frozen at −80 °C for one hour, before proliferation was analyzed using a CyQUANT^®^ Assay Kit (C7026, Life Technologies, Carlsbad, CA, USA). The fluorescence of each well was measured using a microplate reader (Synergy HTX, Biotek Instruments Inc., Winooski, VT, USA) with filters set at EX 485/20 and EM 528/20. A one-way ANOVA was conducted to analyze the difference between hDFa cell treatments.

### 2.5. Wound Healing and Migration Assay

The effects of IAM eluates on the proliferation and migration of hDFa cells (ATCC) were qualitatively assessed using a wound-healing assay. To prepare the amniotic tissue allograft eluates, 2 mL of IAM was centrifugated at 1400× *g* for 15 min, supernatant was discarded, and the sample was resuspended in 1 mL of serum-free DMEM, (Thermo Fisher Scientific, Boston, MA, USA). The centrifugation steps were repeated, IAM eluates were resuspended in 1 mL of serum-free DMEM and incubated at 37 °C with 5% CO_2_ for 48 h. The eluates were filtered using a 0.2 µm polytetrafluoroethylene (PTFE) syringe filter (GE Healthcare, Little Chalfont, UK). The positive control group was treated with DMEM supplemented with 10% FBS (Corning, Waltham, MA, USA), and the negative control group was treated with serum-free DMEM.

For this assay, the cell-culture-treated 96-well plates contained a UV-sterilized, 1 mm width tape (70406, ABC Hobby, Osaka, Japan) on the bottom surface of each well. The 1 mm tape was placed in the middle of each well. Next, hDFa cells were seeded at a density of 30,000 cells/well and incubated for 24 h at 37 °C with 5% CO_2_. After 24 h, the tape was gently removed to create a 1 mm cell free zone into which cells could migrate. The cells were further pre-incubated with 10 µM of mitomycin C (200-008-6, Tocris, Bristol, UK) for 2 h, to inhibit cell proliferation. After the 2 h incubation, the cells were treated with 200 µL of either non-serum DMEM, 10% serum containing DMEM, or IAM eluates in non-serum DMEM. At time point 0, bright field images were captured from three wells to determine the average starting cell-free zone area. At predetermined time points of 12, 24, and 48 h, bright field images were captured from three wells for each condition. Images were analyzed using the ImageJ, version 1.54jwound healing size plugin, and the average starting cell-free zone area was used to calculate the migrated cell area (mm^2^) for each condition at each time point. A 2-way ANOVA was conducted with a Tukey test for multiple comparisons using GraphPad Prism. At the predetermined time points of 0, 12, 24, and 48 h, a LIVE/DEAD viability assay according to the manufacturer’s instructions (Cell Signaling, Denver, MA, USA) was performed. All images were captured using an Olympus IX2 inverted microscope (IX2-SP, Tokyo, Japan).

### 2.6. Endothelial Tube Formation Assay (In Vitro Angiogenesis)

The endothelial tube formation of human umbilical vein endothelial cells (HUVECs) (Gibco) treated with IAM was evaluated by conducting an in vitro angiogenesis assay using an angiogenesis starter kit (A14609-01, Gibco by Life Technologies, New York, NY, USA). HUVECs were cultured in conditioned Medium 200 with large vessel endothelial supplement (LVES) in an incubator set at 37 °C with 5% CO_2_. For this assay, a cell-culture-treated 24-well plate was coated with Geltrex LDEV-Free Reduced Growth Factor Basement Membrane Matrix while cold < 15 °C, and then incubated at 37 °C for at least 30 min to solidify. IAM elution was prepared in triplicate by centrifuging samples at 10,000× *g* for 5 min, removing the supernatant and resuspending the pellet in DMEM without phenol red (A14430-01, Gibco, New York, NY, USA), then placing the samples in a bead bath set to 37 ± 3 °C for 72 ± 1 h. After 72 h, the samples were centrifuged at 10,000× *g* for 5 min, and the elution supernatant was collected. HUVEC suspensions with a concentration of 47,500 cells/250 µL were prepared in the following solutions: a negative control of DMEM without phenol red (BM), a positive control of conditioned Medium 200 (CM), and a IAM elution. Treatments of 47,500 cells/250 µL were added on top of the Geltrex Matrix and incubated for 16 ± 1 h in triplicate for each condition in an incubator set at 37 °C with 5% CO_2_. After incubation, phase contrast images were taken using an Olympus CKX53 inverted microscope. ImageJ with an angiogenesis analyzer plugin was used to conduct a quantitative analysis of the master junctions, master segments, and meshes (tube formations) found in each condition. A one-way ANOVA analysis was conducted using GraphPad Prism.

### 2.7. Human Dermal Fibroblast Cell Viability

The viability of hDFa cells (ATCC) treated with IAM was evaluated by conducting an MTS (CellTiter 96^®^ AQ_ueous_ reagent powder, Promega, Madison, WI, USA) assay on hDFa cells. Cell-culture-treated 96-well plates were used to seed hDFa cells at a cell density of 5000 cells/well in medium supplemented with 10% FBS and 0.01% Pen/Strep. hDFa cells were incubated at 37 °C with 5% CO_2_ for 24 ± 1 h to allow cell adherence prior to treatment. After 24 h, the cell media of 3 wells were aspirated and treated with 120 µL/well of a 20:1 MTS–Phenazine methosulfate (PMS) (98+%, Thermo Fisher, Rockford, IL, USA) mixture in basal media. Similarly, 3 empty blank control wells were treated, and the 96-well plate was incubated at 37 °C with 5% CO_2_ for 1 h (+/−1 min). The absorbance was measured at 490 nm using a microplate reader (Synergy HTX, Biotek Instruments Inc., Winooski, VT, USA). In triplicate, hDFa cells were treated with 200 µL/well of either IAM, vehicle control, supplemented media (complete media as the positive control), or serum-free media (basal media as the negative control), and the 96-well plate was incubated at 37 °C with 5% CO_2_ for 72 ± 1 h. After 72 h, the cell treatments were aspirated from all wells and replaced with 120 µL/well of a 20:1 MTS–PMS mixture in basal media. The 96-well plate was incubated at 37 °C with 5% CO_2_ for 1 h (+/−1 min). The absorbance was measured at 490 nm using a microplate reader (Synergy HTX, Biotek Instruments Inc., Winooski, VT, USA).

### 2.8. Rheometric Properties of the IAM

Three separately manufactured samples of IAM were examined to determine the viscosity of the final product and the rheometric behavior of the fluid through a 22-gauge cannula. To accomplish this, a shear- and temperature-dependent viscosity assay was performed by the Bioengineering Devices Laboratory at Northern Arizona University (NAU). In this assay, the apparent viscosity of IAM was measured with a DHR-2 rotational rheometer with cone-and-plate geometry. For this test, the cone geometry used was a 40 mm-diameter 2° stainless steel cone and the plate was a 40 mm diameter Peltier plate with heating and cooling capabilities. The DHR-2 measured shear rates in reciprocal seconds (1/s) and dynamic viscosity in Pascal seconds (Pa-s).

The samples had been prepared in aliquot volumes according to the DHR-2 hybrid rheometer cone-and-plate specifications. Using a 16G needle, aliquots of 0.6 mL from each sample were aspirated from vials and dispensed onto the rheometer, either with no needle (designated with “neat”) or through a 22G needle (designated with “22G”). For this assay, deionized water was sterile filtered through a 0.22 µm polyethersulfone (PES) filter and used as a negative control. According to the manufacturer’s instructions, the cone was lowered to the specified truncation gap. The Peltier conditioned the temperature of the sample aliquot to 20 °C and soaked at that temperature for 60 s once the temperature had reached equilibrium. The cone geometry then began to shear the sample in a shear sweep from 300 s^−1^ to 600 s^−1^ in increments of 50 s^−1^. The rheometer sheared the sample at each shear rate for 30 s and averaged all data points for each shear rate.

### 2.9. Stability Testing

Stability testing was performed using USP <507> Protein Determination, Method II Bicinchoninic Acid (BCA) Method on one manufactured IAM sample. The IAM sample was tested on the initial manufacture date in 2020 and stored at −80 °C to be tested at each subsequent year over a two year period. The BCA method was carried out using a Pierce^TM^ BCA Protein Assay Kit (23225, Thermo Scientific, Rockford, IL, USA) and 2.0 mg/mL Pierce^TM^ Bovine Serum Albumin Standard (23209, Thermo Scientific, IL, USA) in a 96-well microplate. A standard curve and suitability standard were created using the albumin standard, and all standards and samples were plated in triplicate into the 96-well microplate. A working solution of 50:1 (BCA reagent A: BCA reagent B) was made and added to samples at a 1:8 (sample to working reagent) ratio. The 96-well plate was shaken at 500 rpm for 30 s, followed by incubating at 37 °C for 30 min. The 96-well plate was left at room temperature for 20 min, and the absorbance was measured at 562 nm using an M2 Spectramax plate reader. A one-way ANOVA was performed using GraphPad Prism to compare the three test points.

## 3. Results

### 3.1. Characterization of the Amniotic Fluid Component of IAM by LC-MS/MS Results

LC-MS/MS analysis of the amniotic-fluid-derived portion of the drug substance in IAM contained several extracellular matrix proteins, regulatory proteins, and functional enzymes of interest. The proteins present in the amniotic fluid solids were identified by submitting the raw tandem mass spectral files to SEQUEST software and matching the peptides to known protein sequence databases. A total of 194 proteins were identified by three or more matching peptide sequences. Table 1 lists the extracellular matrix proteins, regulatory proteins, and functional enzymes of interest positively identified in IAM and known to assist in wound healing.

### 3.2. Characterization of IAM by Quantibody Human Cytokine Array Results

The Quantibody Human Cytokine Array conducted on IAM can quantify the concentration of 200 human inflammatory factors, growth factors, chemokines, receptors, and cytokines. A total of 42 different proteins were quantified within the measurable range of the assay. Table 2 lists 12 of the 42 proteins identified by the array. Table 2 lists the class of protein, protein name, average total protein concentrations (in pg/mL), and the protein’s involvement in wound healing.

### 3.3. Hyaluronic Acid Measured in the IAM Results

**Figure 3:**
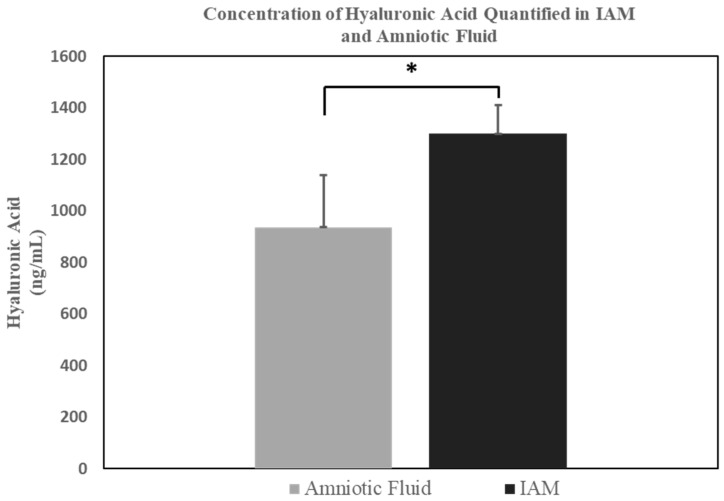
Average concentration of hyaluronic acid in human amniotic fluid and the IAM. n = 3 (* *t*-test, *p*-value < 0.0001).

For comparison, the average concentrations of hyaluronic acid found in three samples of filtered human amniotic fluid and IAM were quantified. The results for the IAM and amniotic fluid samples were interpolated based on the HA standard curve (see Figure 3). The average HA concentration of the filtered amniotic fluid samples was 937 ng/mL with a STD ± 202. The filtered IAM samples contained an average HA concentration of 1299 ng/mL with a STD ± 110. An unpaired *t*-test was conducted, demonstrating a statistically significant difference between the IAM and amniotic fluid.

### 3.4. Human Dermal Fibroblast Cell Proliferation Results

**Figure 4:**
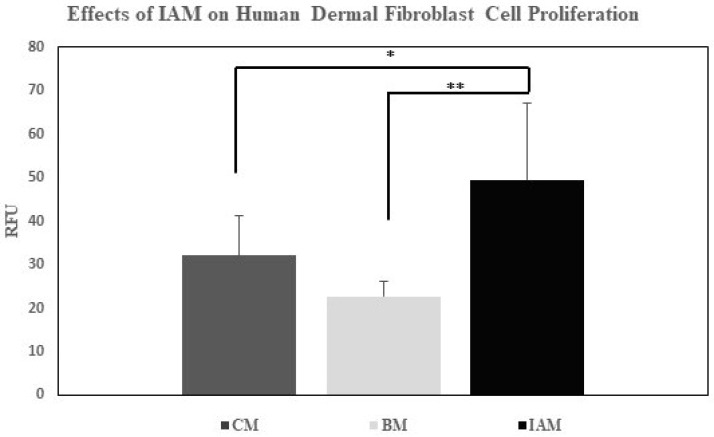
Effect of IAM on the proliferation of hDFa cells. The positive control (CM), negative control (BM), and IAM samples were tested in triplicate and averaged. The RFU measured showed the proliferation of hDFa cells increased when treated with the IAM compared to the negative and positive controls. (* *p*-value < 0.001 ** *p*-value < 0.0001).

To determine the effects of IAM on the cellular proliferation of hDFa cells, a CyQUANT^®^ Cell Proliferation assay was conducted. The relative fluorescence unit (RFU) for each sample was measured using a filter combination for excitation set at 428/20 and emission at 528/20. The average of three CyQUANT^®^ tests was obtained for each cell treatment of either the IAM, supplemented DMEM (complete media as the positive control), or serum-free DMEM (basal media as the negative control). The results were analyzed by one-way ANOVA using GraphPad Prism software (see Figure 4). The complete media positive control measured an average of 32 RFU with a STD ± 9. The basal media negative control measured an average of 23 RFU with a STD ± 3. The cell treatment with IAM measured an average of 57 RFU with a STD ± 19. Statistically significant differences were found between the proliferation of IAM-treated cells and the positive control, with a *p*-value < 0.001. When comparing IAM-treated cells with the negative control, the *p*-value was <0.0001.

### 3.5. Wound Healing and Migration Assy Results

**Figure 5:**
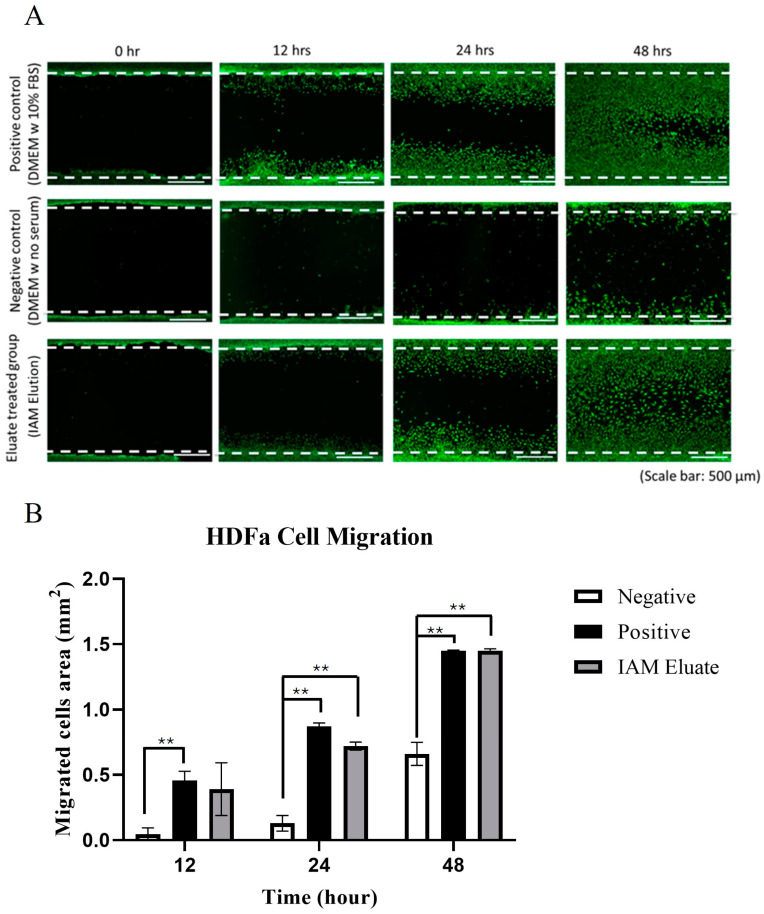
Effect of IAM eluates on the migration of hDFa cells. (**A**) Qualitative representative images of the live cells found in the migration wound-healing assay at various time points (0, 12, 24, and 48 h post-treatment) were obtained. The top row shows the positive control group treated with conditioned DMEM medium, the middle row shows the negative control group treated with serum-free DMEM, and the bottom row shows the group treated with IAM elutes. (**B**) Quantitative analysis of the migrated cell area (mm^2^) for the positive control, negative control, and IAM elutes at time points 12, 24, and 48 h. (** *p*-value ≤ 0.01).

The hDFa cells were divided into three groups: negative control (non-serum DMEM), positive control (conditioned DMEM medium), and IAM eluates. To prevent cell proliferation, all groups were pre-incubated with mitomycin C prior to treatment. Qualitative representative images of the live cells found in the migration assay at various time points (0, 12, 24, and 48 h post-treatment) are shown in Figure 5A. A graph representing the quantitative analysis of the migrated cell area analyzed using the ImageJ wound size plugin is shown in Figure 5B. At time point zero, an average cell-free area of 1.47 mm^2^ was found and used to calculate each condition’s migrated cell area (mm^2^) at each time point. The negative control migrated cell area (mm^2^) was 0.05 ± 0.05 at 12 h, 0.13 ± 0.06 at 24 h, and 0.66 ± 0.09 at 48 h. The positive control migrated cell area (mm^2^) was 0.46 ± 0.07 at 12 h, 0.87 ± 0.02 at 24 h, and 1.45 ± 0.004 at 48 h. The IAM elute treatment migrated cell area (mm^2^) was 0.39 ± 0.20 at 12 h, 0.72 ± 0.03 at 24 h, and 1.45 ± 0.02 at 48 h. A 2-way ANOVA compared treatments with a Tukey multiple comparisons test using GraphPad Prism.

### 3.6. Endothelial Tube Formation Assay (In Vitro Angiogenesis) Results

**Figure 6:**
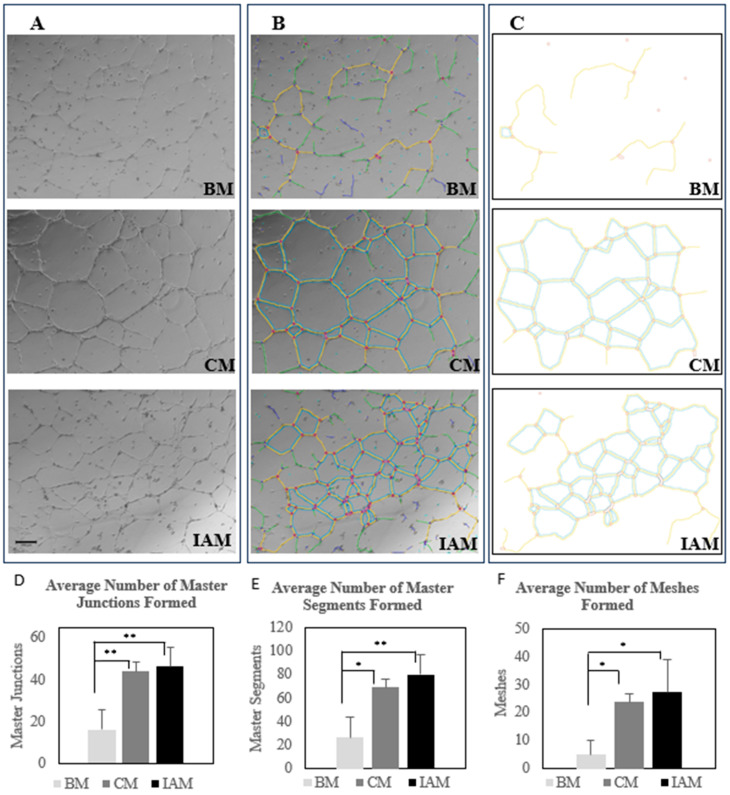
Effect of the IAM on HUVEC tube formation. (**A**) Phase contrast images of negative control BM, positive control CM, and IAM elution. (**B**) Angiogenesis analyzer images of negative control BM, positive control CM, and IAM elution. (**C**) Map images including master junctions (red), master segments (yellow), and meshes (blue) measured by quantitative analysis of negative control BM, positive control CM, and IAM elution. (**D**) Average number of master junctions formed by the negative control BM, positive control CM, and IAM elution. (**E**) Average number of master segments formed by the negative control BM, positive control CM, and IAM elution. (**F**) Average number of meshes (tube formation) formed by the negative control BM, positive control CM, and IAM elution. (* *p*-value < 0.05, ** *p*-value < 0.01).

HUVECs were divided into three groups: negative control (non-serum DMEM (BM), positive control (conditioned Medium 200 (CM), and treatment group (IAM eluates). Phase contrast images analyzed in ImageJ using an angiogenesis analyzer to collect quantitative data on the number of master junctions showed the averages were 16 ± 9.2 in the negative control (BM); 44 ± 4.4 in the positive control (CM); and 46 ± 9.3 in the treatment group (IAM eluate). Master segments had averages of 27 ± 17.2 in the negative control (BM), 69 ± 7.2 in the positive control (CM), and 80 ± 17.3 in the treatment group (IAM elution). Mesh (tube formations) averages were 5 ± 5.3 in the negative control (BM), 24 ± 2.9 in the positive control (CM), and 27 ± 11.7 in the treatment group (IAM elution) (see Figure 6).

### 3.7. Human Dermal Fibroblast Cell Viability Results

**Figure 7:**
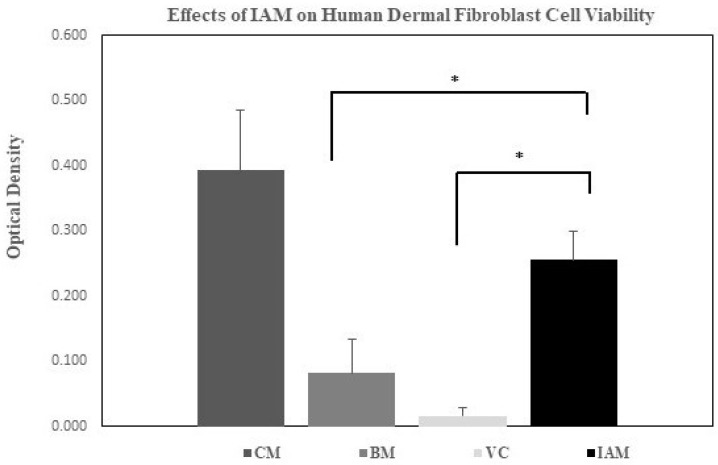
Effect of IAM on hDFa cell viability. The average optical densities of control and IAM-treated hDFa cells were measured to evaluate cell viability. A comparison between the average hDFa cell viability between IAM and either the negative or vehicle control was found to be statistically significant. (* *p*-value < 0.0001).

To determine the effects of IAM on the cellular viability of hDFa cells, a comparison after treatment with either the IAM, supplemented DMEM (complete media as the positive control), serum-free DMEM (basal media as the negative control), or vehicle control was performed by MTS assay. Each treatment was conducted in triplicate and the results were analyzed by one-way ANOVA using GraphPad Prism software (see Figure 7). The average optical densities for the hDFa cell treatments were 0.393 with a STD ± 0.093 when treated with the positive control (CM), 0.082 with a STD ± 0.053 when treated with the negative control (BM), 0.016 with a STD ± 0.014 when treated with the vehicle control (VC), and 0.255 with a STD ± 0.044 when treated with IAM.

### 3.8. Rheometric Properties of the IAM Results

**Figure 8:**
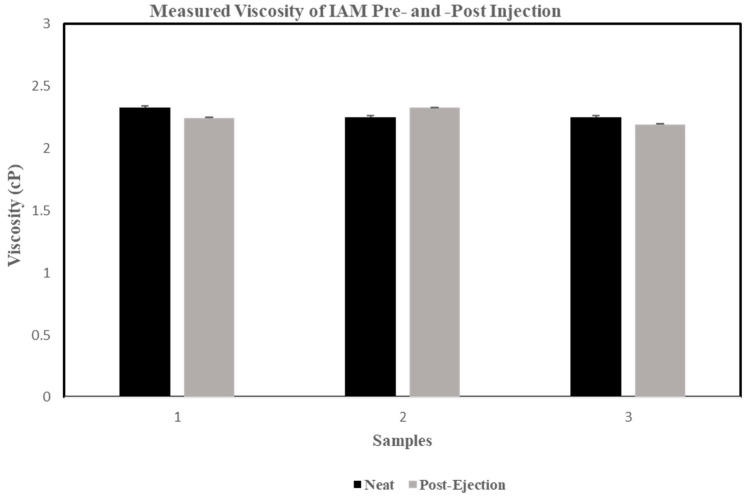
Measured viscosity, in centipoise, of IAM neat solutions and post-injection through a 22-gauge needle. The viscosity of three separately manufactured IAM samples labeled as neat solutions and after the stress of passing through a 22G needle was evaluated. No significant differences were measured.

Viscosity values were evaluated at shear rates between 300 s^−1^ and 600 s^−1^ for all three samples at 20 °C. The shear rate equation was used to determine the viscosity of each sample. For the first sample tested, a viscosity of 2.25 centipoise (Cp) for the neat and a 2.33 Cp for the 22G was measured. The viscosity measured for the second sample was 2.33 Cp neat and 2.24 Cp for the 22G. The third sample tested a viscosity of 2.25 Cp for the neat and a 2.19 Cp for the 22G. An overview of the measured viscosities of all three IAM lots is shown in Figure 8. The viscosity values for each sample and control were within 25% of their respective ranges. The viscosity values for the sterile deionized water control were determined to be within 5% of IAPWS R12-08 values for the viscosity of ordinary water substance. The viscosities were found to be consistent within and across samples of each IAM lot (0.01 Cp or less standard deviation for each sample and less than 0.15 Cp deviation between samples).

### 3.9. Stability Testing Results

**Figure 9:**
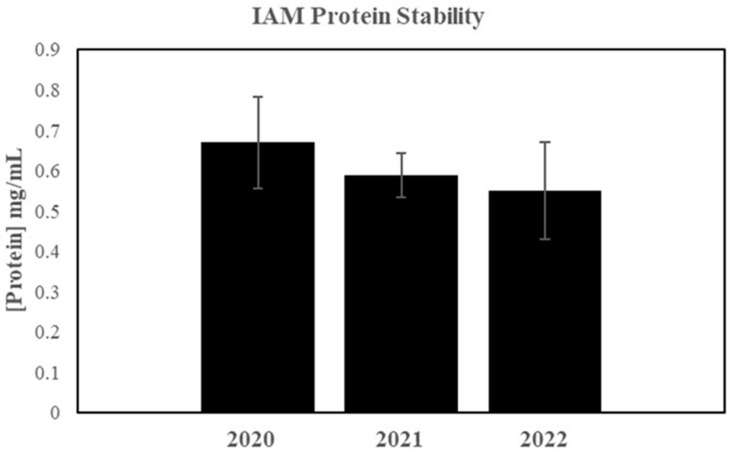
Two-year IAM protein stability test. The protein measurements (in mg/mL) show the stability of the IAM solution when stored at −80 °C. No significant differences were found between the three test points.

The protein concentration was quantified using one manufactured IAM sample at three test points to determine the stability of IAM samples when stored at −80 °C over two years (see Figure 9). The initial average protein concentration in 2020 was 0.67 mg/mL with an STD ± 0.11, the next point in 2021 was 0.59 mg/mL with an STD ± 0.05, and the last point in 2022 was 0.55 mg/mL with an STD ± 0.12. A one-way ANOVA found no significant difference between the three test points.

## 4. Discussion

The number of diabetic diagnoses continues to rise and so does the need to prevent this disease from interfering with everyday life. As previously mentioned, many factors contribute to the delay in DFU wound closure, such as hyperglycemia, vasculopathy, and neuropathy. The absence of a vast network of blood vessels in DFUs prevents the delivery of nutrient-rich oxygenated blood, making them chronic. In wound healing, angiogenesis promotes vessel proliferation and maturation; however, diabetics struggle to maintain this balance. For this purpose, an intracutaneous injectable like an IAM was developed, and in this study, the components and characteristics were assessed for its potential use in the treatment of DFUs.

The components in the IAM were evaluated by mass spectrometry analysis. The amniotic-fluid-derived portion of the drug substance in IAM contains several proteins known to enhance wound healing. As shown in Table 1, a total of 194 proteins were identified by three or more matching peptide sequences of extracellular matrix proteins, regulatory proteins, and functional enzymes of interest in the IAM. For instance, the extracellular matrix proteins fibronectin and vitronectin are found in IAM. Twenty-one matching peptides were detected for fibronectin and three matching peptides for vitronectin. These proteins promote wound healing by facilitating fibroblast–collagen interactions for cell attachment and migration [61]. In the hemostasis stage, fibronectin plays a role in providing structural support for fibrin to bind and stabilize the clot [62,63]. In the inflammatory stage, fibronectin acts as a chemoattractant; meanwhile, in the proliferation and remodeling stages, it promotes re-epithelization [17]. Alterations in vitronectin glycosylation have been associated with an impaired function of endothelial cells observed in patients with hyperglycemia, vascular disease, and diabetes [48]. The fibronectin and vitronectin found in IAM could aid the process of wound healing by promoting cell adhesion, migration, and re-epithelization.

Moreover, three matching peptide sequences of regulatory proteins known as calmodulin and Interleukin-1 receptor antagonist (IL-1Ra) protein were detected in IAM. Calmodulin is a calcium-binding protein that aids in calcium signaling in the skin [64]. Keratinocytes depend on calcium-mediated gradients to proliferate, differentiate, and migrate [64,65]. IL-1Ra has been shown to be suppressed in DM, inhibiting the progression of the inflammatory stage to the proliferative stage by suppressing proinflammatory cytokines and chemokines [66]. IAM can provide IL-1Ra and calmodulin directly to the irregular wound bed of DFUs, facilitating the progression to the next stage of wound healing.

In the IAM, nine matching peptides of plasminogen, a glycoprotein involved in wound healing, cell migration, tissue regeneration, and angiogenesis, were found [67]. Plasminogen has been shown to clear fibrin and decrease inflammation in the later stages of wound healing [68]. Angiotensinogen is an important protein found in IAM, with up to three matching peptide sequences detected. As the sole precursor of all angiotensin peptides, angiotensinogen is a substrate for the renin–angiotensin system, providing blood pressure regulation and homeostasis of sodium and water by means of angiotensin II (Ang II) [69]. The expression of pro-inflammatory and pro-fibrotic cytokines like TGF and TNF can be enhanced by Ang II, facilitating angiogenesis, and promoting keratinocyte and fibroblast migration [50,70,71]. Many studies have shown that inhibition of the proteins found in IAM causes a delay in the efficiency of wound closure and healing.

To determine the concentration of human inflammatory factors, growth factors, chemokines, receptors, and cytokines in IAM, a Quantibody Human Cytokine Array was performed. As shown on Table 2, IAM possesses multiple proteins that promote wound healing by means of angiogenesis, cellular proliferation and migration, ECM regulation, and re-epithelization, to name a few. To illustrate, there is a family of neutral proteases known as matrix metalloproteinases (MMPs) involved in the process of wound healing by regulating inflammation and the degradation of the ECM to facilitate angiogenesis, cellular migration, and tissue regeneration [72]. MMPs are regulated by tissue inhibitor of metalloproteinases (TIMPs), which is responsible for maintaining homeostasis of the ECM during wound healing [52]. Expression of low levels of TIMPs and high levels of MMPs has been observed in DFUs, demonstrating the correlation of the imbalance between MMPs and TIMPs that leads to chronic non-healing ulcers [73]. Many studies have shown TIMP-1 to have a higher affinity for MMP-1 and MMP-9, and TIMP-2 for MMP-2 and MMP-8 [73]. For instance, MMP-1 is involved in keratinocyte migration and re-epithelization, MMP-9 promotes inflammation allowing migration of neutrophils to the wound site, MMP-2 assists with inflammation and keratinocyte migration, and MMP-8 prevents apoptosis and inflammation during wound healing [74]. The TIMP-1 and TIMP-2 found in IAM can be directly administered to DFUs to regulate MMPs, enabling the closure of chronic ulcers and maintaining a balance between TIMPs and MMPs.

Another major component of IAM are growth factors such as insulin-like growth factor binding proteins (IGFBPs), which are known to promote wound healing by binding with high affinity to insulin growth factors (IGFs), IGF-I and IGF-II [56]. A total of six structurally similar IGFBPs (IGFBP-1 to IGFBP-6) have been identified as binding to IGFs for numerous functions; however, some IGFBPs can independently influence cellular proliferation, migration, differentiation, adhesion, and apoptosis [75]. Studies have shown that increased expression of IGF-I can promote re-epithelialization through the migration of keratinocytes to the wound site, in addition to stimulating the rate of hyaluronan synthesis [76,77]. In contrast, high levels of IGFs can negatively contribute to cancer cell growth and other diseases like Alzheimer’s, atherosclerosis, insulin dysregulation, and disorders of the endocrine system [78,79]. For this reason, the regulation of IGFs must be considered when treating chronic wounds like DFUs, as these insulin growth factors play a pivotal role in the wound healing process.

Variable concentrations of IGBP-1, 2, 3, 4, and 6 were identified in the IAM. Several studies have addressed the requirement of IGFBP-1 for IGF-1 to initiate tissue regeneration by facilitating cell adhesion and migration [53,75,80]. IGFBP-2 has an inhibitory effect on IGFs, with higher affinity to IGF-II, and has been demonstrated to be involved in inducing proliferation and migration of human dermal fibroblasts [53]. Studies suggest IGFBP-3 to be ubiquitous in plasma and multiple cell types, binding to plasminogen, vitronectin, fibronectin, and IGF-I at fibrin clots, suggesting the formation of a ternary protein complex indispensable for wound healing [81,82]. Found in IAM and primarily localized in connective tissue is IGFBP-4, a binding protein shown to facilitate cell migration and tendon healing in vivo [55,75,82]. When in combination with vitronectin and IGF-I, both IGFBP-3 and -4 promote cell migration [82]. The matching peptide sequences of fibronectin and vitronectin detected in IAM, along with IGFBPs, can assist in the migration and proliferation of cells at the site of DFUs. Another IGF inhibitor in IAM found to be responsible for assisting in tissue repair and remodeling is IGFBP-6 [56,75]. As an important constituent of the immune system, IGFBP-6 has a variety of roles, such as promoting chemotaxis of monocyte and T-lymphocytes, and including the regulation of hyperthermic responses [56]. The regulation of IGFs to stimulate the re-epithelialization of chronic wounds is an important part of wound management and can be assisted by the administration of IAM.

Another important protein needed for wound closure is osteopontin (OPN), an extracellular matrix protein found in IAM that is responsible for mediating cell migration and adhesion [57]. OPN functions as a cytokine for T-helper 1 cells, in addition to acting as a chemoattractant by stimulating cell-mediated immune responses like B cells and macrophages [83]. Interestingly, OPN also promotes the adhesion and migration of various cell types, such as smooth muscle, endothelial, renal epithelial, and inflammatory cells [84]. The capability of OPN to influence many cells can be observed in its ability to enhance VEGF expression and angiogenesis [84,85,86]. For instance, a key element of angiogenesis is angiogenin (ANG), a ribonuclease found in IAM that functions as a blood vessel homeostasis regulator by promoting the proliferation and migration of endothelial cells [22]. Studies have shown that ANG can regulate the gene expression of other angiogenic factors like VEGF and restore impaired angiogenesis in nonhealing wounds of diabetic mice [87,88]. Moreover, expression of ANG has been identified in the blister fluid of burn wounds, suggesting that ANG is involved in the differentiation of circulating angiogenic cells and in stimulating angiogenesis in vivo [89]. Low levels of ANG have been found in the serum of DFUs, as well as chronic non-healing wounds [87]. These studies suggest that proteins like the OPN and ANG found in IAM are important regulators of VEGF expression and angiogenesis capable of influencing numerous cell types.

Other proteins in IAM involved in wound management include Lipocalin 2 (LCN-2), Growth differentiation factor 15 (GDF-15), and Intercellular Adhesion Molecule-1 (ICAM-1). LCN-2 is also known as neutrophil gelatinase-associated lipocalin, which is an antibacterial and anti-inflammatory protein responsible for transporting small, hydrophobic molecules in the blood stream, modulating cellular growth and metabolism [58]. Many other discoveries about the functions of LCN-2 have been made, but further research is required for a comprehensive understanding. For instance, LCN-2 can degrade the ECM, recruit immune cells to sites of inflammation, and enhance cell proliferation, regulation, and differentiation [58,90]. During inflammation, LCN-2 is upregulated by skin epithelial cells and neutrophils to monitor the immune and inflammatory response and maintain skin homeostasis [90]. As previously mentioned, DFUs remain in the inflammatory phase for long periods of time. A product like IAM can provide and deliver LCN-2 directly to the wound site, to provide modulation of the inflammatory response by facilitating the trafficking of immune cells.

IAM also possesses GDF15, a potential angiogenic cytokine able to enhance VEGF, angiogenesis, and the proliferation of human umbilical vein endothelial cells [91]. During an inflammatory response, expression of GDF15 is upregulated by elevated levels of oxidative stress and hypoxia [92]. Direct delivery of GDF15 to the wound site can decrease the inflammatory response, shortening the healing process of DFUs. Another essential protein found in IAM is ICAM-1, a transmembrane, cell surface glycoprotein expressed in many cells and with a variety of functions that facilitate the process of wound healing [60]. For example, expression of ICAM-1 is induced by many cell types, as it regulates leukocyte trafficking to endothelial cells to initiate an inflammatory response [60,93]. In contrast, ICAM-1 aids in the migration of polymorphonuclear neutrophils to the site of injury, triggering the activation of other proteins to terminate the inflammatory response [94]. Studies have shown that the absence of ICAM-1 during wound healing inhibits keratinocyte migration, angiogenesis, and granulation tissue formation, causing a delay in wound closure [94,95,96]. In this study, we demonstrated multiple human inflammatory factors, growth factors, chemokines, receptors, and cytokines positively identified in IAM that are fundamental in wound management to stimulate and achieve wound closure.

For this purpose, hyaluronic acid has become a GAG of interest, as studies have shown HA to be a key factor in wound healing and skin re-epithelialization during the remodeling stage [97]. HA is known to possess bacteriostatic, biodegradable, and antioxidant properties [98]. Moreover, HA can neutralize oxidative stress, by preventing the cellular damage caused by free radicals [99,100]. This is important, as approximately 50% of HA can be found within the skin, supplying hydration for space filling cavities, improving lubrication of joints, and assisting in cellular proliferation, adhesion, and migration [29]. Furthermore, HA possesses anti-inflammatory properties known to benefit the treatment of diabetic wounds [98]. The concentration of hyaluronic acid was quantified in IAM and compared to human amniotic fluid. These results indicated that IAM is comprised of a higher hyaluronic acid concentration compared to human amniotic fluid. HA can be directly delivered by IAM to support the healing of DFUs through cell proliferation, adhesion, migration, and skin re-epithelization.

To establish the proliferative effects of IAM on hDFa cells, a CyQUANT^®^ assay was performed. This assessment revealed that IAM enhanced the proliferation of hDFa cells compared to the controls established. Upon evaluation of hDFa cell proliferation, a cell viability and wound healing migration assay was conducted to obtain further quantitative and qualitative data on the effects of IAM on hDFa cell viability and migration. Furthermore, the wound healing migration assay showed the positive control group exhibited an increase in migrated cell concentration after 12 h, which continued to rise at the 24 and 48 h time points. Starting at 24 h, a difference in the migration area was observed between eluates and serum-free groups. These results confirmed that IAM eluates promoted the migration of hDFa cells after 24 h. At 48 h, chemotaxis was distinguished, as the cell-free zone was covered by the cells in both the eluate and positive control group. Some cellular migration was observed in the negative control group after 48 h. The data show that the presence of IAM eluates enhanced hDFa cell migration compared to the negative control group. Cells migrating after treatment with IAM eluates demonstrated that hDFa cells were able to proliferate and restore the cellular monolayer. Similarly, a study assessing the migratory effects of human mesenchymal stem cells and proliferative effects of human dermal fibroblast cells treated with dehydrated human amnion/chorion tissue allografts (DHACM) indicated that DHACM promoted both the proliferation and migration of these cell lines compared to the established controls [101].

To verify the potential angiogenic effects of IAM on tube formation using HUVECs, an in vitro angiogenesis assay was conducted. The organized structures analyzed and quantified by ImageJ were master segments, which are segments connected by two master junctions, whose branches are linked with other capillary structures, and meshes, which are closed areas shaped by segments [102]. The results showed no significant difference between any of the analyzed structures when comparing the positive control CM and the IAM elution treatments, but there was a significant difference between the analyzed structures when comparing both the positive control CM and IAM elution against the negative control BM. This confirms that proteins, such as GDF15 and angiogenin, found in IAM can promote angiogenesis and assist in tube formation. Consequently, inadequate angiogenesis highly influences the progress of diabetic wound healing, with raised glucose levels being a root cause of delay [21]. Further studies on the angiogenic effects in high glucose environments would be valuable information to attest if the angiogenic effects of IAM will succeed in a diabetic wound environment. Next, the effects of IAM on hDFa cell viability were assessed by MTS assay. The results indicated that the hDFa cell viability when treated with IAM and either the negative or vehicle control was statistically significant in a one-way ANOVA. Studies have revealed that the cellular activity of epithelial cells when treated with amniotic membrane increases cell viability [103]. Similarly, hDFa cells treated with IAM remained viable, indicating that the population of hDFa cells were healthy.

The rheometric properties of IAM were examined to establish the viscosity of the final product. This test can provide important information of the rheometric behavior of the fluid through a 22-gauge cannula. Hydrodynamic forces like shaking, mixing, and vortexing can cause structural destabilization of proteins to occur [104]. Destabilization of proteins can cause a change in the molecular structure, resulting in protein aggregation, thus affecting the viscosity of the solution [105]. The viscosity levels of an injectable’s solution are associated with the pain perception of the recipient when administered subcutaneously. Depending on the injection site, high viscosity solutions are more tolerable, despite the volume or flow rate of the solution, compared to low viscosity solutions [106]. Although evaluating the solution’s viscosity for pain or discomfort at the injection site is important, this test investigated how the high shear rates experienced by IAM as it passes through a needle affected the sample and provided general information about its viscosity and rheometric properties.

The rheometric data measured with a DHR-2 rotational rheometer demonstrated the viscosity of the product varied negligibly, by less than 0.15 CP, after passing through a 22-gauge space. These results indicate that injecting IAM through a 22-gauge needle does not alter the composition of the final product. In addition to these experiments, stability testing was performed on this drug as per the applicable International Council for Harmonization of Technical Requirements for Pharmaceuticals for Human Use (ICH) and International Organization for Standardization (ISO) standards. The IAM performed well, with real-time shelf life testing at two years when stored at −80 °C. Stability tests were performed using a GLP-validated protocol, and no significant differences were found between all test points over the duration of the validation.

## 5. Conclusions

Diabetes causes significant lifestyle changes, both physical and emotional, which motivate the development of novel treatments like IAM. IAM has the potential to provide rapid wound healing and shorten recovery times by delivering ECM proteins, growth factors, and cytokines directly to the affected site. The results showed that IAM is composed of many proteins shown to promote wound healing through various pathways. This study of IAM highlights its potential as a promising treatment for diabetic foot ulcers in the field of regenerative medicine. It delivers a wide range of proteins and growth factors addressing the critical challenges associated with DFU wound healing, such as poor angiogenesis, delayed cell migration, and chronic inflammation.

The proteins found in IAM—such as fibronectin, vitronectin, plasminogen, and various growth factors—play a crucial role in supporting critical stages of wound healing. For instance, fibronectin and vitronectin are involved in cell adhesion and migration, while plasminogen aids in the breakdown of blood clots. The growth factors, on the other hand, stimulate cell proliferation and differentiation. IAM also contains anti-inflammatory factors like IL-1Ra, which can help regulate the excessive inflammation commonly observed in diabetic wounds. These factors facilitate wound closure and significantly reduce the risk of severe complications, such as foot amputation, highlighting the clinical significance of IAM.

Notably, the in vitro assays that involved culturing hDFa and HUVEC cells with IAM demonstrated significant benefits of IAM in terms of wound healing. These benefits included significant increases in cell viability, migration, and angiogenesis compared to negative controls, further reinforcing its therapeutic potential. Moreover, IAM’s formulation offers stability and a favorable rheological profile, making it suitable for injection without altering its protein composition or efficacy. With real-time stability testing showing positive results, IAM not only demonstrates promise as a long-term solution for diabetic wound management, but the results also provide a sense of reassurance about its potential efficacy over time.

Given the intricate nature of DFUs and the urgent need for effective treatments, IAM offers a novel approach that directly targets the affected site, delivering molecular and cellular factors that benefit wound healing in diabetics. However, to fully establish IAM’s potential for treating chronic wounds in this patient population, further studies modeling the protein interdiffusion in the wound healing process compared to current treatments of DFUs are suggested.

## Figures and Tables

**Table 1:** Proteins identified in the amniotic fluid component of IAM by LC-MS/MS and SEQUEST analysis.

Classification	Protein	Matching Peptides (SEQUEST)	Putative Function in Chronic Wound Healing	Reference
Extracellular Matrix Proteins	Fibronectin	21	ECM adhesive glycoprotein involved in re-epithelialization	[17]
	Vitronectin	3	ECM adhesive glycoprotein aids in promotion of cell adhesion and spreading	[46]
Regulatory Proteins	Calmodulin	3	Mitogen	[47]
	IL-1 Receptor Antagonist Protein	3	Anti-inflammatory	[48]
Functional Enzymes	Plasminogen	9	Precursor to plasmin, degradation of fibrin clots	[49]
	Angiotensinogen	3	Precursor to angiotensin, keratinocyte and fibroblast migration	[50]

**Table 2:** Average concentrations of protein detected by Quantibody Human Cytokine Array in the IAM.

Classification	Protein	Average Protein Conc. (pg/mL)	Putative Function in Chronic Wound Healing	Reference
Matrix Metalloproteinase Inhibitors	TIMP-1	3187	Plays role in ECM composition and wound healing.	[51]
	TIMP-2	2122	Enhances wound healing by stimulating cellular propagation and migration.	[52]
Insulin-like Growth Factor Binding Proteins	IGFBP-1	29,414	Regulates IGF-I in DNA synthesis and wound healing, stimulating hDFa cell migration.	[53]
	IGFBP-2	904	Regulates IGF-II in DNA synthesis and wound healing. Stimulates hDFa cell migration.	
	IGFBP-3	4564	Ubiquitous in plasma, it binds to fibronectin, plasminogen, and IGF-I.	[54]
	IGFBP-4	141,114	Stabilizes IGF-I to stimulate cell differentiation and proliferation.	[55]
	IGFBP-6	671	Regulates cell proliferation, apoptosis, angiogenesis, cell migration, and fibrosis progression.	[56]
Glycophosphoprotein	OPN	3662	Responsible for recruiting inflammatory cells to site of injury. Promotes cell adhesion and migration.	[57]
Ribonuclease	ANG	966	Regulates angiogenesis. Promotes endothelial cell growth, migration, and differentiation.	[22]
Glycoprotein	LCN-2	612	Possesses antibacterial and anti-inflammatory properties. Regulates ECM degradation.	[58]
Transforming growth factor-β superfamily protein	GDF-15	1001	Promotes adaptive angiogenesis.	[59]
Cell surface glycoprotein	ICAM-1	517	Promotes/regulates cell migration and proliferation. Involved in efferocytosis of immune and epithelial cells.	[60]

## Data Availability

The data presented in this study are available on request from the corresponding author due to proprietary trade secrets.

## Abbreviations

AGEs, advanced glycation end products; ECM, extracellular matrix; ROS, reactive oxygen species; PDGF, platelet-derived growth factor; TNF, Tumor Necrosis Factor; TGF, transforming growth factor; VEGF, vascular endothelial growth factor; FGF, fibroblast growth factor; IFN, Interferon; HA, hyaluronic acid; SOC, standard of care; IDSA, Infectious Disease Society of America; WHS, Wound Healing Society; AM, amnion membrane; bFGF, basic fibroblast growth factor; EGF, epidermal growth factor; KGF, keratinocytes growth factor; IL, interleukin; IAM, PalinGen^®^ Flow; HCT/P, Human Cell Tissue Product; hDFa, human Dermal Fibroblast, adult; PTFE, polytetrafluoroethylene; PMS, Phenazine methosulfate; PES, polyethersulfone; BCA, Bicinchoninic Acid assay; HUVECs, human umbilical vein endothelial cells; IL-1Ra, Interleukin-1 receptor antagonist; Ang II, angiotensin II; MMP, matrix metalloproteinases; TIMP, tissue inhibitor of metalloproteinase; IGFBP, insulin-like growth factor binding protein; IGF, insulin growth factor; OPN, Osteopontin; ANG, angiogenin; LCN-2, Lipocalin 2; GDF-15, Growth differentiation factor 15; DhacM, dehydrated human amnion/chorion tissue allografts; ICAM-1, Intercellular Adhesion Molecule-1.
